# The Determinants of Adolescent Glycolipid Metabolism Disorder: A Cohort Study

**DOI:** 10.1155/2022/6214785

**Published:** 2022-06-08

**Authors:** Xiao-Hua Liang, Lun Xiao, Jing-Yu Chen, Ping Qu, Xian Tang, Yuwei Wang

**Affiliations:** ^1^Clinical Epidemiology and Biostatistics Department of Children's Hospital of Chongqing Medical University, National Clinical Research Center for Child Health and Disorders, Ministry of Education Key Laboratory of Child Development and Disorders, Chongqing Key Laboratory of Child Health and Nutrition, Chongqing, China; ^2^Centers for Disease Control and Prevention of Jiulongpo District, Chongqing, China; ^3^Ultrasound Department of Children's Hospital of Chongqing Medical University, Chongqing, China; ^4^Department of Laboratory Medicine, Laboratory for Diagnosis and Treatment of Infectious Diseases Integrated Traditional Chinese and Western Medicine, Chongqing Traditional Chinese Medicine Hospital, Chongqing, China

## Abstract

**Background:**

The increased prevalence of glycolipid metabolism disorders (GLMD) in childhood and adolescents has a well-established association with adult type 2 diabetes and cardiovascular diseases; therefore, determinants of GLMD need to be evaluated during this period.

**Objectives:**

To explore the prevalence of and risk factors for GLMD from the prenatal period through childhood and adolescence.

**Methods:**

A bidirectional cohort study which was established in 2014 and followed between March 1 and July 20, 2019, was used to illustrate the impact factors for GLMD. Stratified cluster sampling in urban-rural areas was used to include subjects from four communities in Chongqing. 2808 healthy children aged between 6 and 9 years in 2014 entered the cohort in 2014 and followed in 2019 with a follow-up rate of 70%. 2,136 samples (aged 11.68 ± 0.60 years) were included.

**Results:**

The prevalence rates of insulin resistance (IR), prediabetes/diabetes, and dyslipidemia were 21.02%, 7.19%, and 21.61%, respectively. Subjects with an urban residence, no pubertal development, dyslipidemia in 2014, higher family income, and higher parental education had significantly elevated fasting insulin (FI) or homeostasis model assessment of insulin resistance (HOMA-IR) levels; subjects with female sex, no pubertal development, dyslipidemia in 2014, obesity, gestational hypertension, maternal weight gain above Institute of Medicine guidelines, and single parents had increased triglyceride or triglyceride/high-density lipoprotein (HDL). Adolescents with rural residence had higher HbA1c level.

**Conclusion:**

We observed that the prevalence of GLMD was high in childhood and adolescents, and rural-urban areas, sex, pubertal development, dyslipidemia in a younger age, maternal obesity, and hypertension were associated with increased GLMD risk, suggesting that implementing the community-family intervention to improve the GLMD of children is essential.

## 1. Background

The increased prevalence of glycolipid metabolism disorders (GLMD) in childhood and adolescents has a well-established association with adult type 2 diabetes and cardiovascular diseases (CVDs) [1]. GLMD in adolescents includes insulin resistance (IR), dyslipidemia, and hyperglycemia. The prevalence of IR and dyslipidemia in children and adolescents ranged from 25.3% to 44.3% among children and adolescents according to different regions and different diagnosed criteria [2, 3]. The triglyceride/high-density lipoprotein cholesterol (HDL-C) ratio was used as an IR marker for overweight and obese children [4] and was also an index of GLMD. The prevalence of hyperglycemia ranged from 5.7% to 11.13% among children with obesity [5]. Despite having lower prevalence than IR and dyslipidemia, hyperglycemia during childhood is a predictor of type 2 diabetes in adulthood [6]. Because childhood metabolic disorders can predict CVDs in adulthood [4, 6], determinants of GLMD need to be evaluated during this period. Therefore, it is meaningful to investigate the prevalence and significant risk factors for GLMD during the perinatal, younger childhood, and adolescence periods.

Obesity is the main cause of GLMD, and our previous study revealed that obesity is positively associated with low-density lipoprotein cholesterol (LDL-C) and TGs but negatively correlated with HDL-C [7]. Moreover, previous studies have shown increased prevalence of GLMD in individuals with a sedentary lifestyle, unhealthy dietary habits, genetic factors, exposure to higher maternal fasting blood glucose (FBG) levels in utero [8], and gestational diabetes [9]. A study found that extraverted personality is positively correlated with triglycerides, FBG, and metabolic syndrome (MS) score in adults [10]. However, to our knowledge, there are no studies from the Southwest of China exploring the correlation between multiple risk factors from prenatal to young adolescent and GLMD in children aged 10∼14 years in a rural-urban cohort study. This cohort study included measures of perinatal variables, social economic status (SES), anthropometric variables, and biochemical indexes in 2014 and 2019 in adolescents, providing an excellent opportunity to fully examine the risk factors for GLMD.

## 2. Methods

### 2.1. Patient and Public Involvement

The children and their guardians or the public were not involved in the design, conduct, reporting, or dissemination plans of our research.

### 2.2. Subjects

**Figure 1:**
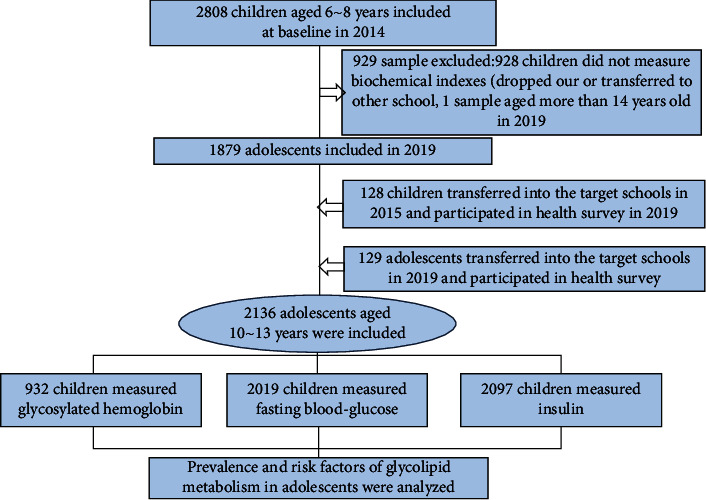
Subjects inclusion process.

Subjects were from a two-stage stratified cluster sampling of urban-rural regions of Chongqing; two streets per county were selected, and, at last, all subjects living in the target region were informed and included in the analyses if they satisfied the following criteria [11–14]. Moreover, a bidirectional cohort in which both retrospective and prospective variables were analyzed to evaluate the risk factors of GLMD from the perinatal period through adolescence, as the variables about perinatal risks were collected, and risk factors and physical examination were conducted in 2014 and in 2019 [11]. Children who had all the following criteria were included: (1) age between six and nine years in 2014, (2) residing in the chosen area for >6 months, (3) did not have severe diseases (e.g., nephropathy, CVD, or cancer), and [15] consent for participation from both the parents and children. The information about SES and family health history questionnaires were collected by a structured questionnaire. The questionnaires were administered and collected by the teachers, and the physical measures results were disseminated also by the teachers. Finally, 2136 participants (with a follow-up rate of 70%) were ultimately included (Figure 1) and the difference between children with follow-up and withdrawal is compared in Supplementary eTable 1.

### 2.3. Demographic Variables

Demographic information and SES (parental occupation, education level, household income, and parent's marriage status) were collected [7, 11, 12, 16, 17]. The education level of parents was measured on a four-point scale (≤9 years (primary and middle school), 9∼12, 12∼15, and >15 years), and we combined bachelor and master's degrees as there were few parents with master's degrees. Prenatal variables included maternal preconception obesity, increased body mass index of mother during pregnancy, birth with Cesarean section, premature delivery (<37 weeks), birthweight, breast-feeding, gestational hypertension (GH), and gestational diabetes. Family history of obesity and CVD was investigated. The degree of pubertal development was surveyed by the visit of pediatrician and children or parents filling the questionnaires, which included the date of the first menstruation and first nocturnal emission, and then the age was calculated.

### 2.4. Physical Examination

Anthropometric indexes were measured by standard-trained pediatric nurses and medical students, and the protocol was detailedly described in our previous papers [11, 14, 18–20]. Anthropometric indexes included height, weight, waist circumference, waist-height ratio (WHtR = waist circumference/height), hip circumference, and blood pressure (BP) [14].

### 2.5. Biochemical Indexes

Venous blood (3 ml) was drawn in the morning after at least 12 hours of fasting from each of the participants who gave informed consents. The biochemical indexes and glycosylated hemoglobin were measured within two hours after venous blood was drawn, which was introduced by several publicized papers [11, 14, 21–23]. Moreover, the ratio of TG/HDL-C was used as a parameter to assess lipid metabolism [4]. Siemens Centaur XP was used to measure fasting insulin (FI), and HbA1c was measured by an automatic hemoglobin analyzer (ARKRAY, Japan).

### 2.6. Diagnostic Criteria

Children were considered to have prediabetes/diabetes if they met at least one of the following criteria: FBG ≥5.6 mmol/L or HbA1c level ≥5.7% [24], and high lipids were defined if adolescents met one of the following criteria [25]: total cholesterol (TC) ≥200 mg/dL, TG ≥ 130 mg/dL, LDL-C ≥ 130 mg/dL, or HDL-C ≤ 40 mg/dL. Moreover, IR was indicated by HOMA-IR > 3.0 based on the criteria from China [2]; HOMA-IR was calculated as (FI mU/L) × (FBG mmol/L)/22.5. Overweight and obesity were diagnosed by a body mass index (BMI) ≥ P_85_ and <P_95_ and BMI ≥ P_95_, respectively, according to the sex-specific Centers for Disease Control BMI-for-age growth charts [26]. Global reference of size for gestational age was used for the diagnosis for large for gestational age (LGA) or small for gestational age (SGA) [27]: birthweight ≥ P_90_ indicated LGA, and birthweight < P_10_ indicated SGA [28], using the mean birthweight of 3,332.93 g and a variation coefficient of 14.36% at 40.5 weeks. Maternal overweight and obesity were indicated by a BMI of 24∼27.9 kg/m^2^ and a BMI ≥ 28 kg/m^2^, respectively; BMI < 18.5 kg/m^2^ was defined as a low BMI [29]. Maternal pregnancy weight gain was diagnosed by the guidelines of the Institute of Medicine (IOM) [30]; the recommendation for underweight, normal weight, overweight, and obese women is to gain 12.5∼18.0 kg, 11.5∼16.0 kg, 7.0∼11.5 kg, and 5·0∼9.0 kg, respectively; if weight gain exceeded that range, weight gain was defined as “above the IOM guidelines”; and if weight gain was below that range, it was defined as “below the IOM guidelines.”

### 2.7. Statistical Analyses

Differences in glycolipid metabolism indexes between two groups were assessed using Student's *t*-test, ANOVA was used to compare more than two groups, and post hoc comparison was performed using Student-Newman-Keuls test. Continuous variables (insulin, HOMA-IR, and TG/HDL) that did not satisfy a normal distribution were subjected to natural logarithmic transformation before analyses. The *χ*^2^ test was used to test the difference in prevalence rates of GLMD. A generalized linear model (GLM) was used to analyze the risk factors that may impact glycolipid metabolism. To reduce the collinearity of variables, model 1 mainly included the variables measured prenatally and in 2014, and model 2 mainly included the variables measured in 2019. Finally, model 3 included all the variables that may impact GLMD. Moreover, multivariable logistic regression was performed using diagnosed GLMD as the dependent variables with the impact factors from perinatal period to adolescence as independent variables. Adjusted *R*^2^ was calculated to reflect the variance of independent variables on dependent variables. Participants with the missing responding variables were not included in the analyses, and the participants who finished the follow-up were compared with those who dropped out.

The data analysis was conducted using SAS 9.4 software (Copyright© 2020 SAS Institute Inc., Cary, NC, USA). A statistical difference was defined by an *α* level of 0.05.

### 2.8. Ethics Approval

All research complied with the ethical guidelines of 1964 Declaration of Helsinki and its later amendments. The Institutional Review Board at the Children's Hospital of Chongqing Medical University approved this study (File no: 2019-86). Informed consent was provided by all subjects and parents/guardians.

## 3. Results

### 3.1. General Characteristics

The general characteristics of the subjects are presented in Table 1. A total of 2,136 samples were included, with a follow-up rate of 70.0%, and the difference of characteristics of childhood between participants with follow-up and withdrawal is described in Supplementary eTable 1. The mean age was 11.68 ± 0.60 years, and 52.25% (1,116/2,136) were males. Biochemical indexes and anthropometric, perinatal, and SES variables are shown in Table 1.

### 3.2. Glycolipid Metabolism of Children with Different Characteristics

Table 2 displays the glycolipid metabolism results in adolescents. Adolescents with the characteristics of urban residence, female sex, older age, no pubertal development, dyslipidemia, and obesity had higher FI or HOMA-IR and TG/HDL than their counterparts. Meanwhile, HbA1c was higher in rural children and those with pubertal development, obesity, or maternal prepregnancy obesity than in their counterparts. In addition, TG/HDL were elevated in children with mother who experienced weight gain above IOM guidelines (*P* < 0.05), single parents (*P* < 0.05), and maternal hypertension (GH) compared with their counterparts (*P* < 0.05 and *P*=0.06). The levels of FI and HOMA-IR were higher in children with parents with higher education levels and family incomes than in their counterparts (*P* < 0.01).

### 3.3. Prevalence of Glycolipid Metabolism Disorder in Adolescents

Table 3 displays the prevalence of childhood GLMD. Overall, the prevalence rates of IR, prediabetes/diabetes, and dyslipidemia were 21.02%, 7.19%, and 21.61%, respectively. The prevalence rates of IR and dyslipidemia were higher in children with the characteristics of older age, dyslipidemia in young childhood (6∼9 years), and obesity than in their counterparts. Moreover, children with urban residence, LGA status, higher family income, and parental education also had increased prevalence of IR. The prevalence of prediabetes/diabetes was higher in children with abdominal obesity in 2014 and maternal prepregnancy obesity than in their counterparts.

### 3.4. Risk Factors of Glycolipid Metabolism Indexes Using a GLM

In GLM 1 (Table 4) (adjusted for sex, age, and region), the results showed that female sex, living in urban areas, and variables measured in 2014 (FBG, BMI, waist circumference [WC]) were risk factors for FI and HOMA-IR levels (all *P* < 0.05), and older age was a risk factor for FI and IR (*P* < 0.01); variables in 2014 (FBG, dyslipidemia, and BMI) were the risk factors for TG/HDL level (all *P* < 0.01), and FBG and BMI in 2014 were risk factors for HbA1c level (Supplementary ETable 2). Model 1 explained 12.43%, 11.92%, 10.32%, and 7.06% of the variance in FI, HOMA-IR, TG/HDL, and HbA1c levels, respectively.

The GLM (Table 4) revealed that female sex, older age, urban residence, and variables in 2019 (higher TG/HDL, BMI, WC, and father's education ≥15 years) were risk factors for FI and HOMA-IR level, whereas increased BMI during pregnancy was a boundary protective factor for FI and HOMA-IR levels (*P*=0.07 and *P*=0.06); HOMA-IR and WHtR in 2019, GH, and maternal weight gain below IOM guidelines were risk factors for TG/HDL levels (all *P* < 0.05), whereas puberty was a protective factor for TG/HDL levels (all *P* < 0.05 or *P* < 0.01); FI in 2019 was a risk factor for HbA1c, and maternal prepregnancy obesity was a borderline risk factor for HbA1c level in model 2 (*P*=0.07) (Supplementary eTable 2). Model 2 explained 26.10%, 24.58%, 17.12%, and 5.90% of the variance in FI, HOMA-IR, TG/HDL, and HbA1c levels, respectively.

Finally, the results of the full model 3 are shown in Table 4. Older age, urban area, FBG in 2014, and variables in 2019 (higher TG/HDL, BMI, WC, and father's education ≥15 years) were significantly correlated with elevated FI and IR levels (all *P* < 0.05), while maternal prepregnancy weight gain was a protective factor for FI and IR levels (all *P* < 0.05). Variables in 2014 (FBG and dyslipidemia) and variables in 2019 (HOMA-IR and WHtR) were risk factors for TG/HDL (all *P* < 0.05). FBG in 2014 and BMI in 2019 were risk factors for HbA1c level (Supplementary eTable 2). The full model explained 28.36%, 26.33%, 19.39%, and 12.33% of the variance of FI, HOMA-IR, TG/HDL, and HbA1c levels, respectively.

### 3.5. Risk Factors for IR, Dyslipidemia, and Prediabetes/Diabetes Based on Logistic Regression

The risk factors for IR, dyslipidemia, and prediabetes/diabetes were analyzed by logistic regression model (Supplementary eTable 3). In the IR model, older age, urban residence, FBG in 2014, BMI in 2019, and father's education ≥15 years had a significant impact on IR prevalence (*P* < 0.05), explaining 20.09% of the variance in IR. The dyslipidemia model showed that single parents, dyslipidemia, high FBG in 2014, and BMI in 2019 were risk factors for dyslipidemia, explaining 12.07% of the variance in dyslipidemia. The prediabetes/diabetes model revealed that WHtR in 2014 was a risk factor for prediabetes/diabetes, explaining 10.29% of the variance in prediabetes/diabetes.

## 4. Discussion

This study is the first bidirectional cohort study from the Southwest of China that involves perinatal, SES, and physical measurements over an average of 12-years' follow-up from prenatal period to adolescence in urban-rural regions to ascertain the prevalence of GLMD and its potential influencing factors. This study found that GLMD was prevalence and the risk factors was from both prenatal and childhood period.

The prevalence of GLMD varies by region and age, and some variance is also attributed to different diagnostic criteria and methods. The current literature describes at least one lipid adverse level prevalence as 19%–25% in US children and adolescents [8, 31], and the prevalence of prediabetes/diabetes in another study [5] was comparable with that of our study. Elevated prevalence of GLMD has been observed in children with obesity in a cross-sectional study [32]. In this study, we found that childhood obesity is the strongest predictor of adolescent GLMD, even when adjusted with other risk factors. Moreover, the prevalence of HOMA-IR exceeded 44% in children who had obesity in comparison with the result from Yin et al.'s study [2], and the prevalence of dyslipidemia reached 28.57% in children with abdominal obesity, suggesting that healthcare programmes should be conducted for children with obesity or abdominal obesity combined with other risk factors.

In addition, a cross-sectional study revealed that elevated TG level was associated with increased HOMA-IR [33], and our cohort study first found that dyslipidemia and elevated fasting glucose at 6∼9 years of age were independent risk factors for HOMA-IR and dyslipidemia in adolescents (10∼14 years old). Adolescents with menarche or spermarche had decreased IR and lipid levels, which indicated that the prepubertal stage will impact GLMD among adolescents. Meanwhile, the transient IR phenomenon emerging during pubertal maturation is accepted as a physiological condition [2], which may be caused by an inadequate *β*-cell response to the decrease in insulin sensitivity [34]. In addition, glycolipid indexes (except HbA1c) were higher in females than in males, which coincided with the results of Interator et al. [35], and the mechanism may be dependent on the difference in the age of prepubertal stages between males and females.

Maternal adverse perinatal experiences will impact GLMD in the offspring [36, 37]. We found that maternal prepregnancy obesity was a risk factor for irregular HbA1c level. An animal study found that maternal obesity permanently alters the hypothalamic response to leptin and subsequently regulates appetite and pancreatic beta-cell physiology [36], which causes maternal and offspring changes in glycolipid levels. Moreover, our study found that both maternal pregnancy weight gain above IOM guidelines and GH were risk factors for elevated offspring TGs, which coincided with the results from young adulthood [38]. This phenomenon can be explained by shared genes or lifestyle. However, the conclusions were controversial, as a study with a small sample size found no association between GH and lipid levels in adolescents [39]; this finding needs to be verified by a large cohort study. In addition, SGA and LGA correlated with elevated HOMA-IR prevalence, which coincided with other findings [40]. Birthweight was correlated with nutritional status in utero, which may cause IR later in life; moreover, LGA is correlated with adolescent obesity, which is essential to IR.

SES is negatively correlated with cardiovascular disease. Our current cohort study provided further support for this concept in the adolescent population. A previous study [41] revealed that marital status of parents was the strongest socioeconomic predictor of young adult arterial stiffness, and we found that the TG level was higher in single-parent adolescents. In addition, the relationship between parental education and the cardiovascular risk of adolescent is controversial, and our results showed a positive relationship between parental education or family income and FI or IR. Studies have revealed a positive correlation between parental education and childhood obesity [42], and obesity was the strongest predictor of insulin sensitivity. Besides, our previous study found that the quality of life and personality traits were significantly associated with metabolic syndrome in children [11]. Moreover, we observed that rural residents have lower FI, IR, and TG levels but higher HbA1c levels, which could be induced by different dietary habits, as rural children consume less fat but more carbohydrates.

There are several limitations in our study. First, as this was a bidirectional cohort study, recall bias may exist for the prenatal variables. Birth certificates were reviewed to verify the birthweight, stature, and gestational age. Second, data on GH and diabetes were collected using a questionnaire, and recall bias existed. However, the perinatal information was collected both in 2014 and in 2019 independently.

In conclusion, the prevalence of GLMD and high glycolipid levels was elevated in adolescents with the features of obesity, maternal prepregnancy obesity, GH, SGA, LGA, and single-parent status. SES was positively correlated with HOMA-IR. To our knowledge, this is the first study to explore the relationship of risk factors from prenatal period to adolescence with glycolipid indexes in a large-sample-size cohort study of adolescents, and the correlation was significant after adjusting for covariates. Our study emphasizes the importance of reducing or controlling adiposity of prepregnancy mother and children, emphasizing the importance of providing support for single-parent children and reducing or preventing GH.

## Figures and Tables

**Table 1:** General characteristics of glycolipid metabolism study in adolescents.

Variables	Participants included in 2019
Sample size	2136
Region	
Urban, no. (%)	1594 (74.63%)
Rural, no. (%)	542 (25.37%)
Anthropometric measures	
Male sex, no. (%)	1116 (52.25%)
Age, mean, y	11.68 (0.60)
BMI, mean, kg/m^2^	19.10 (3.77)
Height, mean, cm	151.78 (7.99)
Weight, mean, kg	44.39 (11.05)
Waist circumference, mean, cm	66.02 (10.14)
WHtR, mean	0.43 (0.06)
Hip circumference, mean, cm	81.80 (8.30)
SBP, mean, mmHg	105.71 (9.56)
DBP, mean, mmHg	62.81 (6.76)
Puberty, no. (%)	586 (31.32%)
Serum biochemical indexes	
FBG, mean, mmol/L	4.45 (0.43)
TC, mean, mmol/L	3.52 (0.61)
TG, mean, mmol/L	1.06 (0.50)
TG, mean^a^	−0.03 (0.39)
HDL-C, mean, mmol/L	1.44 (0.31)
LDL-C, mean, mmol/L	1.84 (0.44)
TG/HDL-C, mean	0.80 (0.50)
Insulin, mean, pmol/L	83.54 (74.85)
Insulin, mean^a^	4.15 (0.73)
HbA1c, mean, %	5.37 (0.19)
Insulin resistance index (IR), mean	2.40 (2.38)
IR, mean^a^	0.57 (0.74)
Uric acid, mean, *μ*mol/L	319.64 (76.98)

Perinatal measures	
Maternal prepregnancy obesity, no. (%)	
Low weight	352 (21.13%)
Normal weight	1158 (69.51%)
Overweight/obesity	156 (9.36%)
Increased BMI during pregnancy, mean, kg/m^2^	5.40 (2.62)
Maternal weight gain, no. (%)	
Weight gain below IOM guidelines	519 (31.36%)
Within IOM guidelines	637 (38.49%)
Weight gain above IOM guidelines	499 (30.15%)
Gestational age of mother, mean, y	27.26 (4.98)
Gestational age of father, mean, y	30.23 (5.31)
Gestational weeks of child, mean, weeks	38.86 (2.16)
Birthweight, mean, g	3271.09 (493.62)
Fatal weight of pregnancy week, no. (%)^b^	
SGA	133 (7.68%)
Appropriate for gestational age	1180 (68.13%)
LGA	419 (24.19)
Gestational hypertension, no. (%)^b^	
No	1967 (97.18%)
Yes	57 (2.82%)
Gestational diabetes, no. (%)^b^	
No	2001 (98.52%)
Yes	30 (1.48%)
Smoking during pregnancy, no. (%)^b^	
No	1642 (87.67%)
Yes	231 (12.33%)
Birth with Cesarean operation, no. (%)^b^	
No	700 (36.76%)
Yes	1204 (63.24%)

Socioeconomic measures	
Income, Yuan/year, no. (%)^*b*^	
∼50,000	645 (31.96%)
∼150,000	853 (42.27%)
>150,000	520 (25.77%)
Expenditure of food, median (IQR), Yuan/month/person	665.6 (499.2, 998.4)
Marriage status, no. (%)^b^	
Double parents	1763 (91.82%)
Single parents	157 (8.18%)
Mother's education, *y*, no. (%)^b^	
∼9	694 (33.27%)
∼12	726 (34.80%)
≥15	666 (31.93%)
Father's education, *y*, no. (%)	
∼9	587 (28.15%)
∼12	750 (35.97%)
≥15	748 (35.88%)
Mother's occupation, no. (%)^b^	
Manager	112 (5.39%)
Worker	708 (34.07%)
Technician/researcher	65 (3.13%)
Farmer	567 (27.29%)
Other	626 (30.13%)
Father's occupation, no. (%)^b^	
Manager	175 (8.49%)
Worker	706 (34.24%)
Technician/researcher	177 (8.58%)
Farmer	573 (27.79%)
Other	431 (20.90%)

^a^Natural logarithmic transformation. ^b^The total sample size is unequal to 2136 in 2019 as there are missing data. BMI: body mass index, WHtR: waist-height ratio, SBP: systemic blood pressure, DBP: diastolic blood pressure, FBG: fasting blood glucose, TC: total cholesterol, TG: triglyceride, HDL-C: high-density lipoprotein cholesterol, LDL-C: low-density lipoprotein cholesterol, IOM: 2009 Institute of Medicine, SGA: small for gestational age, LGA: large for gestational age, QoL: quality of life.

**Table 2:** The glycolipid metabolism levels of adolescent according to perinatal and childhood experiences.

Variables	Insulin, median (IQR)^d^	HOMA-IR, median (IQR)^d^	TG, median (IQR)^d^	TG/HDL, median (IQR)	HbA1c, mean%^e^
Sample size	2097	1979	2018	2018	932
Region					
Urban	60.30 (41.30, 99.40)^f^	1.70 (1.16, 2.79)^f^	0.96 (0.77, 1.24)^f^	0.67 (0.49, 0.96)	5.34 ± 0.19^f^
Rural	55.65 (35.90, 86.50)	1.56 (0.95, 2.34)	0.90 (0.68, 1.22)	0.66 (0.46, 0.96)	5.40 ± 0.18
Anthropometric measures					
Sex					
Male	57.10 (38.20, 93.85)^f^	1.59 (1.05, 2.62)^g^	0.91 (0.70, 1.21)^f^	0.65 (0.46, 0.95)^f^	5.37 ± 0.19
Female	62.00 (42.30, 97.60)	1.71 (1.16, 2.75)	0.98 (0.79, 1.26)	0.69 (0.51, 0.97)	5.37 ± 0.18
Age, y					
∼10	53.50 (35.30, 80.00)^af^	1.53 (1.01, 2.30)^af^	0.91 (0.75, 1.17)	0.64 (0.48, 0.85)^ag^	5.39 ± 0.20
∼11	57.70 (39.90, 91.70)^b^	1.59 (1.09, 2.59)^b^	0.94 (0.74, 1.25)	0.67 (0.48, 0.97)^ab^	5.36 ± 0.19
≥12	67.50 (43.10, 112.40)^c^	1.87 (1.19, 3.13)^c^	0.97 (0.76, 1.26)	0.69 (0.49, 0.98)^b^	5.38 ± 0.18
Pubertal development					
No	60.70 (41.00, 99.40)	1.71 (1.14, 2.83)^f^	0.96 (0.76, 1.26)^f^	0.68 (0.49, 0.98)^g^	5.36 ± 0.20^g^
Yes	59.50 (39.30, 89.40)	1.62 (1.05, 2.55)	0.89 (0.69, 1.17)	0.65 (0.45, 0.92)	5.39 ± 0.18
Dyslipidemia, in 2014					
No	55.4 (37.4, 89.9)^f^	1.59 (1.03, 2.58)^g^	0.91 (0.73, 1.16)^f^	0.62 (0.46, 0.82)^f^	5.37 ± 0.20
Yes	59.2 (41.5, 108.1)	1.64 (1.15, 2.99)	1.02 (0.8, 1.35)	0.78 (0·.56, 1.16)	5.34 ± 0.21
Obesity, in 2014					
Normal	54.60 (36.90, 87.00)^af^	1.52 (1.00, 2.43)^af^	0.92 (0.73, 1.21)^af^	0.64 (0.46, 0.90)^af^	5.37 ± 0.18
Overweight	77.30 (51.20, 124.70)^b^	2.10 (1.42, 3.27)^b^	1.02 (0.84, 1.26)^b^	0.72 (0.59, 1.03)^b^	5.37 ± 0.17
Obesity	89.35 (52.80, 141.90)^b^	2.55 (1.44, 3.91)^b^	1.05 (0.80, 1.42)^b^	0.79 (0.54, 1.13)^b^	5.41 ± 0.20
Obesity, in 2019					
Normal	53.70 (37.20, 82.80)^af^	1.49 (1.02, 2.33)^af^	0.90 (0.72, 1.17)^af^	0.63 (0.46, 0.87)^af^	5.36 ± 0.18^af^
Overweight	79.80 (54.50, 124.65)^b^	2.22 (1.51, 3.39)^b^	1.10 (0.83, 1.39)^b^	0.86 (0.61, 1.15)^b^	5.39 ± 0.17^ab^
Obesity	96.80 (63.65, 150.40)^c^	2.74 (1.78, 4.17)^c^	1.14 (0.91, 1.48)^b^	0.90 (0.66, 1.24)^b^	5.42 ± 0.20^b^
Abdominal obesity, in 2014					
Normal	55.90 (37.80, 90.20)^f^	1.58 (1.03, 2.56)^f^	0.92 (0.73, 1.20)^f^	0.65 (0.47, 0.90)^f^	5.37 ± 0.18
Abdominal obesity	87.50 (52.40, 140.50)	2.36 (1.36, 3.85)	1.03 (0.81, 1.39)	0.79 (0.55, 1.09)	5.40 ± 0.19
Abdominal obesity, in 2019					
Normal	55.60 (38.20, 86.60)^f^	1.55 (1.05, 2.47)^f^	0.91 (0.73, 1.18)^f^	0.64 (0.47, 0.89)^f^	5.36 ± 0.18^f^
Abdominal obesity	90.40 (58.00, 144.70)	2.55 (1·61, 4.05)	1.17 (0.91, 1.50)	0.92 (0.66, 1.27)	5.40 ± 0.20

Perinatal measures					
Maternal prepregnancy obesity					
Low weight	62.20 (41.70, 98.90)	1.71 (1.16, 2.78)	0.95 (0.75, 1.21)	0.66 (0.49, 0.94)	5.36 ± 0.20^abg^
Normal weight	56.00 (39.00, 92.50)	1.58 (1.10, 2.60)	0.96 (0.75, 1.29)	0.70 (0.48, 0.99)	5.34 ± 0.17^a^
Overweight/obesity	61.30 (42.10, 109.30)	1.77 (1.14, 3.08)	1.01 (0.75, 1.23)	0.73 (0.50, 0.96)	5.41 ± 0.16^b^
Maternal pregnancy weight gain					
Below IOM guidelines	62.40 (41.10, 100.30)	1.73 (1.15, 2.73)	0.92 (0.73, 1.17)^ag^	0.65 (0.48, 0.90)	5.37 ± 0.18
Within IOM guidelines	59.65 (39.30, 97.80)	1.67 (1.05, 2.70)	0.97 (0.77, 1.28)^ab^	0.70 (0.49, 0.99)	5.36 ± 0.22
Above IOM guidelines	61.20 (43.00, 96.80)	1.70 (1.18, 2.73)	0.99 (0.75, 1.24)^b^	0.68 (0.51, 0.96)	5.36 ± 0.16
Premature delivery					
No	59.90 (40.50, 96.10)	1.65 (1.13, 2.69)	0.94 (0.75, 1.22)	0.67 (0.48, 0.95)	5.36 ± 0.19
Yes	61.50 (41.20, 104.40)	1.73 (1.12, 2.83)	1.01 (0.77, 1.26)	0.71 (0.49, 1.02)	5.37 ± 0.18
Fatal weight of pregnancy week					
SGA	60.5 (40.4, 94.2)	1.67 (1.13, 2.61)	0.94 (0.74, 1.24)	0.68 (0.48, 0.96)	5.37 ± 0.19
Appropriate for GA	55 (39.3, 85.1)	1.53 (1.04, 2.44)	0.89 (0.73, 1.14)	0.61 (0.44, 0.88)	5.36 ± 0.14
LGA	61.3 (41.6, 105.3)	1.71 (1.15, 3.04)	0.95 (0.76, 1.21)	0.67 (0.49, 0.95)	5.35 ± 0.20
Gestational hypertension					
No	59.90 (40.40, 96.60)	1.67 (1.13, 2.70)	0.94 (0.75, 1.24)	0.67 (0.48, 0.96)	5.37 ± 0.19
Yes	59.70 (40.50, 88.55)	1.62 (1.12, 2.61)	0.99 (0.76, 1.32)	0.70 (0.54, 1.06)	5.35 ± 0.20
Gestational diabetes					
No	59.90 (40.20, 96.60)	1.66 (1.12, 2.70)	0.94 (0.75, 1.24)	0.67 (0.48, 0.96)	5.37 ± 0.19
Yes	73.00 (46.60, 90.20)	1.92 (1.21, 3.00)	1.01 (0.74, 1.22)	0.70 (0.55, 0.97)	5.41 ± 0.17
Birth with Cesarean operation					
No	57.40 (38.50, 93.90)	1.60 (1.08, 2.61)	0.93 (0.76, 1.23)	0.67 (0.49, 0.96)	5.36 ± 0.20
Yes	60.60 (41.55, 96.85)	1.69 (1.15, 2.74)	0.96 (0.75, 1.25)	0.68 (0.48, 0.96)	5.37 ± 0.18
Breast-feeding					
No	65.20 (40.50, 106.00)	1.78 (1.13, 2.90)	0.95 (0.8, 1.24)	0.69 (0.54, 0.95)	5.38 ± 0.18
Yes	59.50 (39.10, 96.20)	1.62 (1.07, 2.72)	0.92 (0.72, 1.23)	0.65 (0.46, 0.96)	5.38 ± 0.19

Socioeconomic measures					
Income, Yuan/year					
∼50,000	56.20 (38.35, 90.00)^af^	1.57 (1.07, 2.55)^af^	0.96 (0.75, 1.26)	0.66 (0.48, 1.00)	5.38 ± 0.20
∼150,000	60.50 (40.00, 94.80)^a^	1.64 (1.07, 2.64)^a^	0.93 (0.76, 1.21)	0.66 (0.48, 0.93)	5.36 ± 0.19
>150,000	63.85 (42.60, 106.45)^b^	1.79 (1.19, 3.09)^b^	0.94 (0.73, 1.22)	0.66 (0.48, 0.96)	5.38 ± 0.17
Marriage status					
Double parents	59.90 (40.40, 96.20)	1.67 (1.13, 2.70)	0.94 (0.75, 1.24)^g^	0.67 (0.48, 0.96)	5.37 ± 0.19
Single parents	61.90 (40.10, 91.20)	1.67 (1.11, 2.59)	1.06 (0.80, 1.30)	0.72 (0.51, 1.05)	5.35 ± 0.22
Mother's education, y					
∼9	56.10 (36.90, 93.50)^af^	1.59 (0.97, 2.55)^af^	0.94 (0.73, 1.25)	0.66 (0.47, 0.97)	5.37 ± 0.18
∼12	58.20 (40.40, 92.60)^ab^	1.62 (1.14, 2.62)^ab^	0.93 (0.76, 1.23)	0.66 (0.49, 0.96)	5.38 ± 0.20
≥15	63.85 (43.60, 100.25)^b^	1.76 (1.22, 2.90)^b^	0.96 (0.75, 1.23)	0.68 (0.49, 0.95)	5.36 ± 0.20
Father's education, y					
∼9	54.85 (36.90, 90.60)^af^	1.52 (0.97, 2.48)^af^	0.93 (0.74, 1.22)	0·65 (0.48, 0.94)	5.38 ± 0.19
∼12	58.95 (40.50, 92.00)^a^	1.62 (1.15, 2.65)^a^	0.94 (0.74, 1.22)	0.66 (0.47, 0.96)	5.38 ± 0.18
≥15	64.40 (42.35, 105.70)^b^	1.80 (1.17, 3.00)^b^	0.97 (0.76, 1.26)	0.69 (0.50, 0.97)	5.36 ± 0.20
Mother's occupation					
Manager	71.90 (43.10, 127.80)	1.93 (1.16, 3.27)	1.00 (0.75, 1.31)	0.75 (0.48, 1.03)	5.39 ± 0.22
Worker	60.50 (39.80, 92.60)	1.67 (1.09, 2.66)	0.95 (0.74, 1.26)	0.67 (0.49, 0.97)	5.38 ± 0.18
Technician/researcher	62.50 (45.50, 92.65)	1.73 (1.38, 2.72)	1.01 (0.7, 1.19)	0.68 (0.46, 0.84)	5.33 ± 0.15
Farmer	57.60 (39.70, 96.70)	1.64 (1.09, 2.70)	0.93 (0.75, 1.26)	0.66 (0.48, 0.98)	5.36 ± 0.19
Other	58.20 (40.00, 96.90)	1.61 (1.13, 2.65)	0.94 (0.76, 1.19)	0.67 (0.47, 0.92)	5.37 ± 0.19
Father's occupation					
Manager	65.60 (41.90, 96.85)	1.81 (1.17, 2.78)	0.94 (0.77, 1.24)	0.68 (0.50, 0.91)	5.40 ± 0.20
Worker	58.65 (38.90, 89.90)	1.62 (1.05, 2.55)	0.94 (0.74, 1.23)	0.66 (0.49, 0.97)	5.37 ± 0.19
Technician/researcher	61.10 (39.50, 116.30)	1.69 (1.07, 3.39)	0.96 (0.73, 1.26)	0.69 (0.46, 0.96)	5.34 ± 0.23
Farmer	57.00 (40.10, 94.80)	1.62 (1.14, 2.67)	0.94 (0.75, 1.28)	0.67 (0.48, 0.98)	5.38 ± 0.19
Other	60.05 (40.70, 99.50)	1.69 (1.16, 2.83)	0.94 (0.76, 1.17)	0.66 (0.49, 0.95)	5.37 ± 0.16

^a,b,c^Difference of post hoc analyses among groups; different letters mean the difference existed between two groups. ^d^Natural logarithmic transformation was used to calculate the *P*value. ^e^932 samples were included. ^f^*P* < 0.01; ^g^*P* < 0.05. SGA: small for gestational age, GA: gestational age, LGA: large for gestational age.

**Table 3:** The prevalence of glycolipid metabolism for adolescent according to perinatal and childhood experiences.

Variables	HOMA-IR (>3)		Dyslipidemia		Prediabetes	
	Prevalence	*P*	Prevalence	*P*	Prevalence	*P*
Sample size	416 (21.02%)		436 (21.61%)		67 (7.19%)	
Region						
Urban	336 (22.86%)	<0·01	309 (20.7%)	0.09	28 (6.91%)	0.78
Rural	80 (15.72%)		127 (24.19%)		39 (7.40%)	
Anthropometric measures						
Sex						
Male	205 (19.86%)	0.19	223 (21.2%)	0.64	41 (8.17%)	0.21
Female	211 (22.28%)		213 (22.05%)		26 (6.05%)	
Age, y						
∼10	44 (15.17%)	<0·01	49 (16.55%)	0.03	7 (6.67%)	0.74
∼11	205 (19.51%)		252 (23.53%)		39 (7.80%)	
≥12	167 (26.18%)		135 (20.74%)		21 (6.42%)	
Pubertal development						
No	276 (23.08%)	<0·01	262 (21.56%)	0·96	34 (7.80%)	0.69
Yes	96 (17.55%)		120 (21.47%)		29 (7.09%)	
Dyslipidemia, in 2014						
No	138 (18.42%)	0·06	114 (15.64%)	<0·01	28 (8.75%)	0.12
Yes	61 (23.74%)		96 (32.65%)		5 (4.31%)	
Obesity, in 2014						
Normal	200 (17.33%)	<0·01	242 (20.46%)	<0·01	34 (6.19%)	0.06
Overweight	56 (29.63%)		44 (22.92%)		6 (6.19%)	
Obesity	73 (38.42%)		60 (31.41%)		16 (11.85%)	
Obesity, in 2019						
Normal	245 (16.21%)	<0·01	281 (18.33%)	<0·01	41 (6.35%)	0.15
Overweight	89 (32.36%)		86 (30.71%)		6 (5.94%)	
Obesity	81 (44.75%)		64 (34·78%)		18 (10.47%)	
Abdominal obesity, in 2014						
Normal	243 (19.57%)	<0·01	265 (20.88%)	0.05	39 (6.20%)	0.02
Abdominal obesity	64 (32.99%)		53 (26.9%)		15 (11.90%)	
Abdominal obesity, in 2019						
Normal	287 (17.28%)	<0·01	321 (19.04%)	<0·01	45 (6.27%)	0.07
Abdominal obesity	127 (41.91%)		108 (35.06%)		20 (9.95%)	

Perinatal measures						
Maternal prepregnancy obesity						
Low weight	241 (22.25%)	0.12	221 (20.16%)	0.06	36 (7.33%)	0.02
Normal weight	57 (17.87%)		84 (26.09%)		3 (2.17%)	
Overweight/obesity	37 (25.52%)		35 (23.33%)		9 (11.69%)	
Maternal pregnancy weight gain						
Below IOM guidelines	105 (22.01%)	0.98	96 (19.92%)	0.45	16 (7.21%)	0.55
Within IOM guidelines	129 (21.57%)		140 (23.1%)		22 (7.64%)	
Above IOM guidelines	100 (21.65%)		102 (21.75%)		10 (5.15%)	
Premature delivery						
No	317 (21.40%)	0.53	316 (21%)	0.12	47 (6.98%)	0.79
Yes	44 (23.40%)		49 (25.93%)		5 (6.17%)	
Fatal weight of pregnancy week						
SGA	226 (20.68%)	0.03	236 (21.22%)	0.72	35 (7.09%)	0.91
Appropriate for GA	20 (15.75%)		24 (18.75%)		4 (5.88%)	
LGA	99 (25.52%)		87 (22.14%)		13 (7.43%)	
Gestational hypertension						
No	384 (21.03%)	0.83	399 (21.45%)	0.48	61 (7.30%)	0.85
Yes	12 (22.22%)		14 (25.45%)		2 (8.33%)	
Gestational diabetes						
No	393 (21.14%)	0.62	409 (21.59%)	0.63	63 (7.35%)	0.37
Yes	7 (25.00%)		5 (17.86%)		0 (0.00%)	
Birth with Cesarean operation						
No	132 (20.06%)	0.34	139 (20.81%)	0.51	22 (6.92%)	0.87
Yes	245 (21.99%)		250 (22.12%)		35 (7.22%)	
Breast-feeding						
No	43 (23.24%)	0.55	46 (24.6%)	0.40	5 (5.32%)	0.47
Yes	207 (21.27%)		217 (21.83%)		42 (7.38%)	

Socioeconomic measures						
Income, Yuan/year						
∼50,000	114 (18.84%)	0.02	146 (23.78%)	0.21	27 (8.39%)	0.51
∼150,000	161 (20.43%)		160 (19.88%)		24 (6.47%)	
>150,000	122 (25.42%)		105 (21.47%)		12 (6.09%)	
Marriage status						
Double parents	349 (21.33%)	0.47	356 (21.42%)	0.44	56 (7.49%)	0.30
Single parents	28 (18.79%)		36 (24.16%)		3 (4.17%)	
Mother's education, y						
∼9	125 (19.20%)	0.05	153 (22.97%)	0.41	24 (6.02%)	0.43
∼12	132 (19.76%)		145 (21.45%)		25 (8.59%)	
≥15	149 (24.31%)		125 (19.94%)		17 (7.56%)	
Father's education, y						
∼9	102 (18.44%)	<0.01	128 (22.78%)	0.44	23 (6.69%)	0.74
∼12	134 (19.20%)		157 (22.11%)		26 (8.12%)	
≥15	170 (25.00%)		139 (19.97%)		17 (6.77%)	
Mother's occupation						
Manager	29 (27.36%)	0.61	32 (29.91%)	0.09	6 (11.32%)	0.81
Worker	135 (20.58%)		133 (19.79%)		21 (6.58%)	
Technician/researcher	13 (21.31%)		10 (16.39%)		2 (8.00%)	
Farmer	110 (20.87%)		126 (23.55%)		20 (7.49%)	
Other	119 (20.70%)		121 (20.65%)		17 (6.88%)	
Father's occupation						
Manager	35 (22.15%)	0.09	33 (20.50%)	0.55	7 (10.29%)	0.53
Worker	130 (19.55%)		136 (20.09%)		28 (8.31%)	
Technician/researcher	47 (29.19%)		32 (19.39%)		5 (8.62%)	
Farmer	106 (19.78%)		129 (23.71%)		17 (6.46%)	
Other	84 (21.37%)		89 (22.19%)		9 (5.03%)	

^a, b ,c^Difference of post hoc analyses among groups; different letters mean the difference existed between two groups. ^d^Natural logarithmic transformation was used to calculate the *P*value. ^e^932 samples were included. ^f^*P* < 0.01; ^g^*P* < 0.05. SGA: small for gestational age, GA: gestational age, LGA: large for gestational age.

**Table 4:** The risk factors for glycolipid indexes levels in adolescents.

Variables	Insulin, pmol/L			HOMA-IR level			TG, mmol/L			TG/HDL		
	*β*	*P*	*R* ^2^	*β*	*P*	*R* ^2^	*β*	*P*	*R* ^2^	*β*	*P*	*R* ^2^
Model 1: variables in 2014												
Sex, male *versus* female	−0.136	0.004	12,43%	−0.110	0.029	11.92%	−0.112	<0.001	6.79%	−0.102	<0.001	10.32%
Age, y	0.149	<0.001		0.123	0.005		0.026	0.21		0.041	0.12	
Region, urban *versus* rural	0.213	0.005		0.285	<0.001		0.108	<0.001		0.074	0.12	
Prepregnancy weight gain, kg/m^2^	−0.008	0.425		−0.011	0.299		—	—		—	—	
Birthweight, 50 g	−0.001	0.644		−0.002	0.439		−0.002	0.19		−0.002	0.21	
FBG in 2014, mmol/L	0.14	0.005		0.142	0.006		0.080	<0.001		0.099	<0.001	
Dyslipidemia in 2014	0.064	0.238		0.044	0.450		0.099	<0.001		0.218	<0.001	
BMI in 2014, kg/m^2^	0.031	0.018		0.041	0.003		0.019	<0.001		0.035	<0.001	
Waist in 2014, cm	0.018	<0.001		0.017	0.002		—	—		—	—	
Gestational hypertension	—	—		—	—		0.096	0.20		0.093	0.33	

Model 2: variables in 2019												
Sex, male *versus* female	−0.187	<0.001	26.10%	−0.168	<0.001	24.58%	−0.08	<0.001	16.00%	−0.091	<0.001	17.12%
Age, y	0.136	<0.001		0.124	<0.001		−0.011	0.51		0.001	0.98	
Region, urban *versus* rural	0.147	0.001		0.214	<0.001		−0.04	0.15		−0.091	0.01	
Prepregnancy weight gain, kg/m^2^	−0.012	0.071		−0.013	0.059		—	—		—	—	
Birthweight, 50 g	−0.001	0.612		−0.001	0.618		0.001	0.89		0.001	0.80	
HOMA-IR level in 2019^a^	—	—		—	—		0.144	<0.001		0.179	<0.001	
TG/HDL in 2019	0.293	<0.001		0.288	<0.001		—	—		—	—	
BMI in 2019, kg/m^2^	0.033	<0.001		0.036	<0.001		—	—		—	—	
Waist in 2019, cm	0.014	<0.001		0.014	<0.001		—	—		—	—	
WHtR in 2019	—	—		—	—		1.150	<0.001		1.901	<0.001	
Gestational hypertension	—	—		—	—		0.139	0.02		0.157	0.04	
Prenatal weight gain												
Below IOM guidelines	—	—		—	—		—	<0.001		0.069	0.02	
Above IOM guidelines	—	—		—	—		0.037	0.13		0.033	0.29	
Puberty development	—	—		—	—		−0.083	<0.001		−0.072	0.03	
Father's education, ref. ≤9 y												
9∼12	0·085	0·040		0·081	0·070		—	—		—	—	
≥15	0·183	<0·001		0·177	<0·001		—	—		—	—	

Model 3: full model												
Gender, male *versus* female	−0.178	<0.001	28.36%	−0.159	<0.001	26.33%	−0.107	<0.001	17.67%	−0.094	<0.001	
Age, y	0.135	<0.001		0.117	0.001		−0.013	0.58		−0.009	0.76	
Region, urban *versus* rural	0.230	0.001		0.296	<0.001		0.010	0.85		−0.024	0.71	
Prepregnancy weight gain, kg/m^2^	−0.019	0.020		−0.019	0.025		—	—		—	—	
Birthweight, 50 g	−0·001	0.495		−0.002	0.459		−0.001	0.39		−0.002	0.40	
FBG in 2014, mmol/L	0.124	0.003		0.125	0.005		0.070	<0.001		0.098	<0.001	
Dyslipidemia in 2014	—	—		—	—		0.055	0.06		0.168	<0.001	
TG/HDL in 2019	0.271	<0.001		0.261	<0.001		—	—		—	—	
BMI in 2019, kg/m^2^	0.045	<0.001		0.049	<0.001		—	—		—	—	
Waist in 2019, cm	0.012	0.009		0.011	0.023		—	—		—	—	
BMI in 2014, kg/m^2^	—	—		—	—		−0.008	0.22		−0.001	0.93	
HOMA-IR level in 2019^a^	—	—		—	—		0.146	<0.001		0.17	<0.001	
WHtR in 2019	—	—		—	—		1.272	<0.001		1.687	<0.001	
Gestational hypertension	—	—		—	—		0.168	0.05		0.174	0.13	
Prenatal weight gain												
Below IOM guidelines	—	—		—	—		0.051	0.11		0.058	0.15	
Above IOM guidelines	—	—		—	—		0.059	0.08		0.078	0.07	
Puberty	—	—		—	—		−0.076	0.11		−0.081	0.18	
Education, ref. ≤9 y												
9∼12	0.081	0.123		0.08	0.145		—	—		—	—	
≥15	0.180	0.001		0.165	0.003		—	—		—	—	

^a^Natural logarithm transformation. FBG: fasting blood glucose, BMI: body mass index, IR: insulin resistance, TG/HDL-C: the triglyceride/high-density lipoprotein cholesterol (HDL-C) ratio, WHtR: waist-height ratio, IOM: 2009 Institute of Medicine.

## Data Availability

The data used to support the findings of this study were supplied by Xiaohua Liang and cannot be made freely available. Requests for access to these data should be made to [Xiaohua Liang, xiaohualiang@hospital.cqmu.edu.cn].

## Supplementary Materials

Supplementary Materials eTable 1: general characteristics of childhood between participants with follow-up and withdrawal. eTable 2: the risk factors for HbA1c level in adolescents. eTable 3: the logistic regression model of IR and glycolipid metabolism disorder.
