# Supplementary material for: The Determinants of Adolescent Glycolipid Metabolism Disorder: A Cohort Study
Source: Int J Endocrinol. 2022 Jun 8;2022:6214785. doi: 10.1155/2022/6214785 (PMC9200567; doi:10.1155/2022/6214785)
Supplement: Supplementary Materials — eTable 1: general characteristics of childhood between participants with follow-up and withdrawal. eTable 2: the risk factors for HbA1c level in adolescents. eTable 3: the logistic regression model of IR and glycolipid metabolism disorder. [file 6214785.f1.zip › 6214785.f1/Supplementary_etable_1_(8.2) (1).docx]

| **eTable 1: General Characteristics of Childhood Between Participants with Follow-up and Withdraw** | | | |
| --- | --- | --- | --- |
| **Variables** | **Participants followed up** | **Withdraw samples** | ***P*** |
| **Sample size** | 1879 | 929 |  |
| **Region** |  |  |  |
| Urban | 1337(71·15%) | 776(83·53%) | <0·01 |
| Rural | 542(28·85%) | 153(16·47%) |  |
| ***Anthropometric measures*** |  |  |  |
| Sex, male (n (%)) | 980(52·16%) | 494(53·18%) | 0·61 |
| Age, y | 7·27±0·62 | 7·24±0·62 | 0·25 |
| BMI, kg/m^2^ | 16·7±2·76 | 16·81±3·31 | 0·38 |
| Height, cm | 124·81±6·15 | 124·47±6·48 | 0·20 |
| Weight, kg | 26·21±5·8 | 26·21±6·08 | 0·99 |
| Waist circumference, cm | 56·18±7·31 | 55·71±7·08 | 0·12 |
| WHtR | 0·45±0·05 | 0·45±0·05 | 0·22 |
| SBP, mmHg | 100·24±8·74 | 99·87±8·77 | 0·30 |
| DBP, mmHg | 62·59±7·85 | 62·18±7·24 | 0·18 |
| HR | 98·93±13·76 | 99·97±13·75 | 0·07 |
| ***Serum biochemical indexes*** | |  |  |
| FBG, mmol/L | 4·12±0·59 | 4·11±0·58 | 0·67 |
| TC, mmol/L | 3·54±0·72 | 3·58±1·85 | 0·51 |
| tg, mmol/L | 1·18±9·53 | 0·98±1·26 | 0·45 |
| hdl-C, mmol/L | 1·27±0·27 | 1·25±0·27 | 0·09 |
| ldl-C, mmol/L | 1·74±0·57 | 2·37±11·45 | 0·14 |
| **Gestational hypertension** ***^a^*** |  |  |  |
| No | 1729(97·08%) | 658(98·95%) | 0·01 |
| Yes | 52(2·92%) | 7(1·05%) |  |
| **Gestational diabetes** *^b^* |  |  |  |
| No | 28(98·43%) | 653(97·32%) | 0·07 |
| Yes | 28(1·57%) | 18(2·68%) |  |
| ***Socioeconomic measures*** |  |  |  |
| **Income, Yuan/month/person** *^c^* |  |  |  |
| ~500 | 68(4·61%) | 25(3·68%) | 0·43 |
| ~1,000 | 98(6·64%) | 41(6·04%) |  |
| ~2,000 | 248(16·8%) | 95(13·99%) |  |
| ~3,000 | 307(20·8%) | 155(22·83%) |  |
| ~5,000 | 378(25·61%) | 183(26·95%) |  |
| >5,000 | 377(25·54%) | 180(26·51%) |  |
| *^a^* with 362 missing data; *^b^* with 349 missing data; *^c^* with 659 missing data. | | | |
| BMI: body mass index; WHtR: waist-height-ratio; SBP: systemic blood pressure; DBP: diastolic blood pressure; HR: heart rate; FBG: fasting blood glucose; TC: total cholesterol; TG: triglyceride; HDL-C: high density lipoprotein cholesterol; LDL-C: low density lipoprotein cholesterol. | | | |
