# Supplementary material for: The Determinants of Adolescent Glycolipid Metabolism Disorder: A Cohort Study
Source: Int J Endocrinol. 2022 Jun 8;2022:6214785. doi: 10.1155/2022/6214785 (PMC9200567; doi:10.1155/2022/6214785)
Supplement: Supplementary Materials — eTable 1: general characteristics of childhood between participants with follow-up and withdrawal. eTable 2: the risk factors for HbA1c level in adolescents. eTable 3: the logistic regression model of IR and glycolipid metabolism disorder. [file 6214785.f1.zip › 6214785.f1/Supplementary_etable_2_(8.2) (1).docx]

| **eTable 2: The Risk Factors of HbA1c Level in Adolescents** | | | | |
| --- | --- | --- | --- | --- |
| **Variables** | ***β*** | **SE** | ***P*** | **R^2^** |
| **Model 1: Variables in 2014** |  |  |  |  |
| Sex, male *vs.* female | -0·004 | 0·019 | 0·85 | 7·06% |
| Age, y | 0·001 | 0·016 | 0·99 |  |
| Region, Urban *vs.* Rural | 0·011 | 0·025 | 0·66 |  |
| FBG in 2014, mmol/L | 0·074 | 0·019 | <0·001 |  |
| BMI in 2014, kg/m^2^ | 0·007 | 0·003 | 0·01 |  |
| **Model 2: Variables in 2019** |  |  |  |  |
| Sex, male *vs.* female | -0·001 | 0·015 | 0·941 | 5·90% |
| Age, y | -0·006 | 0·012 | 0·614 |  |
| Region, Urban *vs.* Rural | -0·060 | 0·017 | 0·001 |  |
| Insulin in 2019, pmol/L | 0·025 | 0·011 | 0·029 |  |
| WHtR in 2019 | 0·193 | 0·118 | 0·103 |  |
| Puberty | 0·016 | 0·017 | 0·354 |  |
| Maternal prepregnancy obesity, ref. normal |  |  |  |  |
| Low weight | -0·011 | 0·018 | 0·539 |  |
| Overweight/Obesity | 0·042 | 0·023 | 0·073 |  |
| **Model 3: Full model** |  |  |  |  |
| Sex, male *vs.* female | -0·001 | 0·022 | 0·978 | 12·33% |
| Age, y | 0·006 | 0·019 | 0·730 |  |
| Region, Urban *vs.* Rural | -0·005 | 0·031 | 0·864 |  |
| FBG in 2014, mmol/L | 0·084 | 0·020 | <0·001 |  |
| Insulin in 2019, pmol/L | 0·017 | 0·016 | 0·289 |  |
| BMI in 2019, kg/m^2^ | 0·450 | 0·176 | 0·011 |  |
| Puberty | -0·009 | 0·028 | 0·742 |  |
| Maternal prepregnancy obesity |  |  |  |  |
| Low weight *vs.* normal | 0·010 | 0·027 | 0·726 |  |
| Overweight/Obesity *vs.* normal | 0·054 | 0·035 | 0·119 |  |
| FBG: fasting blood glucose; BMI: body mass index; WHtR: waist-to-height ratio. | | | | |
