# Supplementary material for: The Determinants of Adolescent Glycolipid Metabolism Disorder: A Cohort Study
Source: Int J Endocrinol. 2022 Jun 8;2022:6214785. doi: 10.1155/2022/6214785 (PMC9200567; doi:10.1155/2022/6214785)
Supplement: Supplementary Materials — eTable 1: general characteristics of childhood between participants with follow-up and withdrawal. eTable 2: the risk factors for HbA1c level in adolescents. eTable 3: the logistic regression model of IR and glycolipid metabolism disorder. [file 6214785.f1.zip › 6214785.f1/Supplementary_etable_3_(8.2) (1).docx]

| **eTable 3: The Logistic Regression Model of IR and Glycolipid Metabolism Disorder** | | | |
| --- | --- | --- | --- |
| **Variables** | **OR (95% CI)** | ***P*** | **R^2^** |
| ***Insulin resistance model*** |  |  |  |
| Sex, male *vs.* female | 0·74(0·49,1·1) | 0·1378 | 20·10% |
| Age, y | 1·39(0·98,1·97) | 0·0632 |  |
| Region, Urban *vs.* Rural | 3·45(1·74,6·86) | 0·0004 |  |
| Increased BMI during pregnancy, kg/m^2^ | 0·94(0·86,1·02) | 0·1094 |  |
| Dyslipidemia in 2014 | 1·21(0·79,1·86) | 0·3805 |  |
| FBG in 2014, mmol/L | 1·47(0·98,2·22) | 0·0622 |  |
| BMI in 2019 | 1·25(1·18,1·32) | <·0001 |  |
| Father's Education, ref. ≤9y |  |  |  |
| 9~12 | 1·50(0·86,2·61) | 0·7356 |  |
| ≥15 | 1·95(1·12,3·39) | 0·0322 |  |
| Gestational hypertension | 0·47(0·09,2·34) | 0·3556 |  |
| ***Dyslipidaemia model*** |  |  |  |
| Sex, male *vs.* female | 0·93(0·64,1·36) | 0·7053 | 12·07% |
| Age, y | 0·89(0·64,1·24) | 0·4882 |  |
| Region, Urban *vs.* Rural | 1·20(0·68,2·13) | 0·5345 |  |
| Maternal weight gain during pregnancy, kg/m^2^ | 0·94(0·87,1·01) | 0·0958 |  |
| Gestational hypertension | 1·21(0·37,3·93) | 0·7525 |  |
| Birth weight, 50g | 1·00(0·98,1·02) | 0·7000 |  |
| Dyslipidemia in 2014 | 2·62(1·77,3·86) | <·0001 |  |
| FBG in 2014, mmol/L | 1·74(1·18,2·59) | 0·0057 |  |
| BMI in 2019 | 1·10(1·05,1·16) | 0·0001 |  |
| Marriage status, Double parents *vs.* Single parents | 1·53(0·83,2·81) | 0·1759 |  |
| ***Prediabetes model*** |  |  |  |
| Sex, male *vs.* female | 1·66(0·71,3·88) | 0·2400 | 10·29% |
| Age, y | 0·47(0·23,0·93) | 0·0300 |  |
| Region, Urban *vs.* Rural | 1·08(0·41,2·87) | 0·8800 |  |
| Birth weight, 50g | 1·03(0·99,1·07) | 0·1300 |  |
| FBG in 2014, mmol/L | 1·37(0·67,2·78) | 0·3900 |  |
| WHtR in 2019 | 1·05(1·01,1·11) | 0·0400 |  |
| IR: Insulin resistance; FBG: fasting blood glucose; BMI: body mass index; WHtR: waist-to-height ratio. | | | |
